# The European Hernia Society Prehabilitation Project: A Systematic Review of Intra-Operative Prevention Strategies for Surgical Site Occurrences in Ventral Hernia Surgery

**DOI:** 10.3389/fsurg.2022.847279

**Published:** 2022-07-13

**Authors:** D. Wouters, G. Cavallaro, Kristian K. Jensen, B. East, B. Jíšová, L. N. Jorgensen, M. López-Cano, V. Rodrigues-Gonçalves, C. Stabilini, F. Berrevoet

**Affiliations:** ^1^ Department for General and HPB Surgery and Liver Transplantation, University Hospital Gent, Gent, Belgium; ^2^ Department of Surgery “P. Valdoni”, Sapienza Unviersity, Rome, Italy; ^3^ Digestive Disease Center, Bispebjerg Hospital, University of Copenhagen, Copenhagen, Denmark; ^4^ 3rd Department of Surgery and 1st Medical Faculty of Charles University, Motol University Hospital, Prague, Czech Republic; ^5^ Abdominal Wall Surgery Unit, Department of General Surgery, Hospital Universitari Vall d’Hebron, Universitat Autònoma de Barcelona, Barcelona, Spain; ^6^ Department of Surgery, University of Genoa, Genoa, Italy; ^7^ European Hernia Society, Ospedale Policlinico San Martino IRCCS, Genoa, Italy

**Keywords:** surgical site occurrence, surgical site infection, abdominal wall repair, hernia, prevention

## Abstract

**Background:**

Ventral hernia repair is one of the most commonly performed surgical procedures worldwide. To reduce the risk of complications, pre- and intra-operative strategies have received increasing focus in recent years. To assess possible preventive surgical strategies, this European Hernia Society endorsed project was launched. The aim of this review was to evaluate the current literature focusing on pre- and intra-operative strategies for surgical site occurrences (SSO) and specifically surgical site infection (SSI) in ventral hernia repair.

**Methods:**

A systematic review was conducted and reported in line with the Preferred Reporting Items for Systematic Reviews and Meta-Analyses statement. Databases used were Pubmed and Web of Science. Original retrospective or prospective human adult studies describing at least one intra-operative intervention to reduce SSO after ventral hernia repair were considered eligible.

**Results:**

From a total of 4775 results, a total of 18 papers were considered suitable after full text reading. Prehospital chlorhexidine gluconate (CHG) scrub appears to increase the risk of SSO in patients undergoing ventral hernia repair, while there is no association between any type of surgical hat worn and the incidence of postoperative wound events. Intraoperative measures as prophylactic negative pressure therapy, surgical drain placement and the use of quilt sutures seem beneficial for decreasing the incidence of SSO and/or SSI. No positive effect has been shown for antibiotic soaking of a synthetic mesh, nor for the use of fibrin sealants.

**Conclusion:**

This review identified a limited amount of literature describing specific preventive measures and techniques during ventral hernia repair. An advantage of prophylactic negative pressure therapy in prevention of SSI was observed, but different tools to decrease SSIs and SSOs continuously further need our full attention to improve patient outcomes and to lower overall costs.

## Introduction

Treatment of abdominal wall hernias is an imperative and rapidly evolving field of general surgery. Difficulty of the repair varies according to the indication and type of procedure, from low-risk repair of primary hernias to high-risk abdominal wall reconstruction (1, 2). Due to its frequent use of prosthetic material, prevention of wound complications is essential to avoid long-term mesh-related infection and hernia recurrence (3, 4). Furthermore, wound morbidity after hernia surgery increases hospital costs and significantly reduces patient-reported quality of life (5, 6).

Besides the fact that abdominal wall surgery requires specific attention and a tailored approach towards prevention of tissue healing complications, a standardized definition for reporting wound morbidity is mandatory. So far, several different systems have been used, including the ones put forth by the Centers for Disease Control and Prevention, the American College of Surgeons National Surgery Quality Improvement Program, the Ventral Hernia Working Group and the Clavien-Dindo classification system. Haskins et al. proposed to standardize the reporting of ventral hernia related wound events to surgical site infection (SSI), surgical site occurrence (SSO) and SSO requiring procedural intervention (SSOPI) (7). SSI reflects an infection that occurs in the part of the body where the surgery took place and is defined further on the compartment involved, whereas SSO includes any SSI as well as any other wound healing issue, e.g. wound cellulitis, skin or soft tissue ischemia or necrosis, skin or subcutaneous tissue dehiscence, fascial disruption, exposed mesh, hematoma, seroma as well as wound serous drainage.

In general, SSI remains a frequent and challenging complication of surgery. SSI accounts for greater than 20% of all health care associate infections, pneumonia being the most common nosocomial infection (8).

The prevention of postoperative wound complications after abdominal wall repair is multifactorial and several initiatives have been initiated as clinical practice guidelines and the development of SSI-prevention bundles (9–11).

Recent literature findings show a close relation between tissue healing complications and hernia recurrence, further highlighting the importance of their prevention (12, 13).

Recent literature tends to -rightfully so- lay focus on the importance of patient-specific prehabilitation for surgery, including adequate management of underlying systemic comorbidities, lifestyle and chronic medical therapy. This is in fact the subject of a parallel data analysis conducted by this research team (14). However, besides patient-specific prehabilitation, general prevention strategies that can be applied to most or all patients with ventral hernia to provide optimal standard of care should be implemented as well.

The objective of this paper is to review common and emerging intra-operative SSO prevention strategies in ventral hernia repair, discussing concrete preventive measures for the surgical team to use in the operating theatre to improve patient outcome after abdominal wall repair.

## Methods

The World Health Organization (WHO) has published extensive guidelines regarding prevention of surgical site infection/occurrence for general surgery (15). A systematic review was conducted to include all new literature on the prevention of SSI/SSO since the WHO publication in 2016, now limited to hernia repair. Recent literature suggested that incisional versus primary ventral hernia patient populations vary too much to pool their data (16, 17). However, since the scarcity of hernia-specific literature on the subject did not allow for the desired separate analysis of primary and incisional ventral hernia, evidence quality was downgraded according to indirectness. This review uses the WHO guidelines as a reference value for all literature predating 2016. For prevention methods not covered by the 2016 WHO publication, the most recent systematic review was identified and discussed as comparison.

### Search Strategy

**Figure 1:**
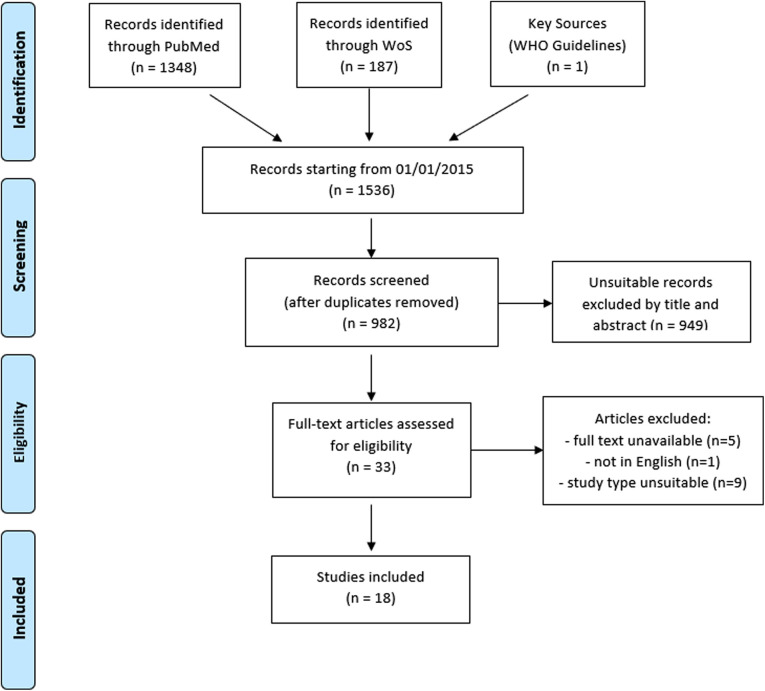
PRISMA flow chart.

This systematic review was conducted and reported in line with the Preferred Reporting Items for Systematic Reviews and Meta-Analyses (PRISMA) statement (18) (Figure 1). Databases used were Pubmed and Web of Science.

The search string in free text search contained following keywords:

“hernia” AND “prevention” AND (“surgical site infection” OR “dehiscence” OR “SSI” OR “seroma” OR “hematoma” OR “necrosis” OR “SSO” or “surgical site occurrence”).

Additionally, a Pubmed MESH term search was conducted using the following string:

“Hernia, Ventral"[Mesh] AND (“Surgical Wound Dehiscence"[Mesh] OR “Surgical Wound Infection"[Mesh] OR “Seroma"[Mesh] OR “Hematoma"[Mesh] OR “Postoperative Complications"[Mesh] OR “Herniorrhaphy/adverse effects"[Mesh]).

### Eligibility Criteria

Manuscript types considered eligible for inclusion were original retrospective or prospective human adult studies describing at least one intra-operative intervention to reduce SSI/SSO after ventral hernia repair, not related to hernia repair technique or mesh type. A date limitation was set for articles from 01/01/2015 on. The last search was performed on 01 November 2021. Only articles written in English were included. Articles were excluded if the full text was unavailable. Due to the heterogeneity of papers included, and an expected paucity of randomized controlled trials, no meta-analysis was planned. This review was initiated on behalf of and endorsed by the European Hernia Society.

### Data Extraction and Outcome

All identified citations were screened by an individual researchers (DW), sequentially reviewing title, abstract and finally full text. Where there was uncertainty for inclusion, this was discussed with the senior researcher (FB).

### Risk of Bias

The risk of bias was evaluated for all included studies. The Cochrane Collaboration tool for assessment of the risk of bias was applied to RCTs. For cohort studies, risk of bias was assessed by using the Newcastle–Ottawa quality assessment scale (NOS).

## Results

From a total of 4775 results, a total of 35 suitable papers were identified for full text review. After full text screening, 18 studies were considered suitable and included in this systematic review (Table 1). For the sake of clarity, each result section will be preceded by a short narrative introduction on the prevention measure in question. An overview of results is given in Table 2, with a separate overview for the more numerously studied subject of NPWT (see Table 3).

**Table 1:** Included studies with characteristics, study endpoints and follow-up.

Measure	Author	Year	Study design	Outcome variables			Minimal FU (months)
				SSI	SSO	Recurrence	
CHG scrub	Prabhu (19)	2017	Retrospective	Yes	Yes	No	1
Surgical hat	Haskins (20)	2017	Retrospective	Yes	Yes	No	1
Mesh Notouch	Schneeberger (21)	2020	Retrospective	Yes	Yes	Yes	12
AB soaking	Yabanoglu (22)	2015	RCT	Yes	Yes	No	3
Drains	Westphalen (23)	2015	RCT	Yes	Yes	No	1
	Plymale (24)	2016	Retrospective	Yes	Yes	No	4 (median)
	Wong (25)	2016	Retrospective	Yes	No	No	1
	Krpata (26)	2017	Retrospective	Yes	Yes	No	1
pNPWT	Gassman (27)	2015	Retrospective	Yes	Yes	Yes	3
	Rodriguez (28)	2015	Retrospective	Yes	Yes	Yes	3
	Soares (29)	2015	Retrospective	Yes	Yes	Yes	3
	De Vries (30)	2017	Retrospective	Yes	Yes	Yes	3
	Licari (31)	2020	Retrospective	Yes	Yes	No	3
	Hopkins (32)	2020	Retrospective	Yes	Yes	Yes	1
	Bueno-Lledo (33)	2020	RCT	Yes	Yes	No	1
Cauterization	Prassas (34)	2018	Retrospective	No	Yes	Yes	18
Quilt sutures	Alhussini (35)	2019	RCT	No	Yes	No	1
Fibrin Sealant	Azoury (36)	2015	Retrospective	Yes	Yes	Yes	1

**Table 2:** Overview of results-all excluding NPWT.

Measure	Author	Year	Study type	*N*	Objective	Endpoint	Significant findings
Prehospital CHG scrub	Prabhu	2017	Retrospective	3924	To determine whether preoperative chlorhexidine gluconate decreases the risk of 30-day wound morbidity in patients undergoing ventral hernia repair	SSO SSI	The preoperative chlorhexidine scrub group had a higher incidence of SSOs (odds ratio [OR] = 1.34; 95% CI 1.11 to 1.61) and SSIs (OR = 1.46; 95% CI 1.03 to 2.07). Prehospital chlorhexidine gluconate scrub appears to increase the risk of 30-day wound morbidity in patients undergoing ventral hernia repair.
Surgical hat	Haskins	2017	Retrospective	6210	To investigate the association between type of surgical hat worn by surgeons and the incidence of postoperative wound events following ventral hernia repair	SSO SSI	The type of surgical hat worn by surgeons was not found to be associated with an increased risk of 30-day surgical site infections or surgical site occurrences requiring procedural intervention.
No-touch	Schneeberger	2020	Retrospective	88	To evaluate the use of a “no-touch” technique with antibiotic solution during synthetic mesh placement in ventral hernia repairs and its impact on complication/infection rates	Short-term (<30 days), Medium-term (30 to 90 days), and long-term (91 to 365 days) complications	Fourteen patients (15.9%) experienced postoperative complications. A total of 16 complication events occurred in the cohort (two patients had multiple complications): 13 short-term complications (81.3 percent), three medium-term complications (18.7 percent), and zero long-term complications. The authors conclude that the no-touch technique for mesh placement in ventral hernia repairs appears to be efficacious in minimizing infectious complications with mesh placement.
AB soaking	Yabanoglu	2015	RCT	52	To investigate the effect of the use of synthetic mesh soaked in vancomycin solution on the rate of graft infection	SSO SSI	Seroma development was significantly more common in group 2 (*P* < 0.041). Three patients (5.7%) developed superficial wound infection, and 9 (17%) developed surgical site infection 2-type wound-site infection. No significant difference was found between the groups in terms of infection. The use of synthetic mesh soaked in vancomycin solution had no beneficial effects on the rate of wound-site infection.
Drains	Westphalen	2015	RCT	42	To compare the incidence of seroma and surgical wound infection between patients subjected to large incisional hernia repair by means of the onlay technique, with one group being subjected to the placement of drains, while progressive tension sutures without drains were used in a second group	Seroma SSI	The occurrence of seroma was not significantly different between groups (*p* 0.469; 0.631; 0.619). Surgical wound infection occurred 19% in group 1 and 23.8% in group 2, without a significant difference between the groups (*p* > 0.999). The frequency of seroma and infection did not exhibit significant differences between individuals subjected to onlay mesh repair of large incisional hernias with drains or progressive tension sutures without drainage.
	Plymale	2016	Retrospective	18	To ascertain if the number of days postoperatively that drains are left in place impacts the incidence of surgical site complications	SSO SSI	No significant relationship was found between incidence of seroma/hematoma and days postoperatively of last drain removal. Wound complications increased linearly with drain time. Only body mass index >35 remained an independent predictor of wound occurrence, *P* < 0.05. Wound complications occur frequently after AWR. Wound infections occur more commonly among patients with drains in place for more than 2 weeks.
	Wong	2016	Retrospective	234	To determine whether the use of extended postoperative antibiotic prophylaxis beyond standard Surgical Care Improvement Project guidelines with closed-suction surgical drain placement in incisional ventral hernia repair reduces the incidence of postoperative surgical-site infections	SSI	Extended postoperative prophylactic antibiotics significantly reduce the incidence of postoperative surgical-site infections (OR, 0.31; *p* < 0.01). As the hernia grade increased, the odds ratio tended to decrease, suggesting that extended prophylactic antibiotics may be more effective at decreasing the incidence of surgical-site infections at higher grades. Extended antibiotic prophylaxis reduces surgical site infection risk following complex ventral hernia repairs, and should be considered in all cases.
	Krpata	2017	Retrospective	581	To investigate the impact of retromuscular drains on SSO following retromuscular VHR with synthetic mesh	SSO SSI SSOPI	Retromuscular drains were less likely to develop a noninfectious SSO (OR, 0.33). Drain placement was not associated with SSI (OR, 1.30) or SSOPI (OR, 0.94). Based on an analysis of early outcomes, surgical drains do not increase the risk of surgical infectious complications, and may be protective against some SSOs, such as seroma formation.
Cauterization	Prassas	2018	Retrospective	94	To investigate the effectiveness of cauterization of the hernia sac in terms of reducing the incidence of postoperative seroma formation after standard laparoscopic intraperitoneal mesh repair without closure of the central defect (sIPOM)	Seroma Postoperative pain Recurrence rate	The cauterization group had significantly lower rate of seroma formation, compared to the control [0 vs. 25% (*n* = 5), *p* < 0.05]. There was no difference noted regarding postoperative pain between the two techniques. Hernia recurrence rate was found to be higher in the control group [0 vs 12.5% (*n* = 2), *p* < 0.05]. Electric cauterization of the hernia sac significantly reduces the rate of postoperative seroma compared to standard laparoscopic repair in patients with ventral and incisional hernias.
Quilt sutures	Alhussini	2019	RCT	370	To evaluate using quilting sutures in a prospective randomized controlled manner the decrease in the incidence of seroma formation among patients subjected to ventral hernia repair	Seroma	There was significantly smaller amount of output of the drains in every day of the first five postoperative days as well as the total amount of the output before drain removal in favor of the quilting group. Drains were removed earlier in group B. The incidence of clinically detected seroma was less in group B as well.
Fibrin sealant	Azoury	2015	Retrospective	250	To evaluate the ability of a fibrin sealant to reduce the incidence of post-operative seroma following abdominal wall hernia repair	SSO	Surgical site occurrences occurred in 18.1% of the TISSEEL and 13% of the non-TISSEEL group (*P* = 0.27). There was a trend towards an increased incidence of seroma in the TISSEEL group (TISSEEL 11%, non-TISSEEL 4.9%, *P* = 0.07). A total of $124,472.50 was spent on TISSEEL, at an average cost of $995.78 per case. In the largest study to date, TISSEEL™ application offered no advantage for the reduction of post-operative seroma formation following complex abdominal hernia repair. Moreover, the use of this sealant was associated with significant costs.

**Table 3:** Overview of results (NPWT).

Author	Year	Study design	*N*	Objective	Endpoint	Significant findings
Gassman	2015	Retrospective	61	To examine whether primary wound events were different between patients who had primary closure with NPT versus patients who only had primary closure after AWR	Recurrence rate	The application of NPWT leads to lower hernia recurrence rate of 25 versus 3% and significant reduction of SSI rate (17 versus 5 cases, *p* = 0.01). The distribution of wound infections was different with the control group having more deep tissue and organ space infections than the NPWT group. The numbers are too small to make any real conclusions from this data.
Rodriguez	2015	Retrospective	117	To evaluate whether the NPWT would improve surgical site outcomes following VHR in patients with grade 3 hernias.	Recurrence rate SSO SSI	SSO rates compare favorably with reported historical 30-day SSOs rates for high-grade ventral hernias, which range between 39 and 55%. Use of the NPWT system may lead to decreased postoperative complications in an extremely high-risk patient population.
Soares	2015	Retrospective	199	To assess the impact of a modified negative-pressure wound therapy system (hybrid-VAC or HVAC) on outcomes of open VHR.	Recurrence rate SSI SSO Length of stay	The NPWT cohort had lower surgical site infections (9% vs 32%, *P*, 0.001) and surgical site occurrences (17% vs 42%, *P* 5.001) rates. The HVAC group had a significantly longer LOS in hospital with 84% of patients in the HVAC group incurring a hospitalization of 7 days or more versus 39% of the SWD group (*P* < .001). The NPWT system is associated with optimized outcomes following open VHR.
De Vries	2017	Retrospective	66	Evaluation of NPWT in the reduction of wound infections and other wound complications in **high-risk patients** undergoing **major cVWHR**	SSO SSI	NPWT was associated with a significant decrease in incisional wound infection rates (48 versus 7% (*p* = 0.01, OR 0.08 (95% CI 0.16–0.39)). Opening of the wound (spontaneous or interventional) was significantly decreased after the introduction of pNPWT (7 versus 48%, *p *<* *0.001, OR 0.08 (95% CI 0.02–0.39)). No reduction of other wound complications was seen.
Bueno-Lledo	2020	RCT	146	Evaluation of NPWT in the reduction of surgical site occurrences (SSOs) and the length of stay after incisional hernia repair	SSO Length of stay	Significatively higher incidence of SSOs in the control group compared to the treatment group (29.8% vs 16.6%, *P* < 0.042). There was no SSI in the treatment group and 6 cases in the control group (0% vs 8%, *P* < 0.002). No significant differences regarding seroma, hematoma, wound dehiscence, and length of stay were observed between the groups
Hopkins	2020	Retrospective	85	Determining the effect of NPWT on the incidence of SSI after **complex** incisional hernia repair	SSO SSI Length of stay	NPWT was associated with significantly lower rates of deep SSI (2.9% vs. 17.6%, *p* = 0.045) Median LOS was longer in patients with iNPWT (7 vs. 5 days, *p* = 0.001).
Licari	2020	Retrospective	180	To compare the post-operative outcomes of **at risk patients** who underwent VHR when treated with standard wound care vs NPWT and to perform a spending review	SSO SSI Cost effectiveness Length of stay	Nine (12.8%) patients in the NPWT group and 48 (43.6%) in the control group developed a wound complication (*p* < 0.0001, RR 0.29 (0.15–0.56)), suggesting that infection is less likely to occur in NPWT-treated incisions, compared with standard wound care. This study demonstrates that NPWT use in high-risk populations following VHR is associated with positive clinical and economic outcomes.

### Prehospital Chlorhexidine Gluconate Scrub (PCS)

While not strictly intra-operative, this prevention strategy was included because of its general nature as opposed to patient-specific. Prabhu et al. retrospectively analyzed 3924 ventral hernia patients from the AHSCQ data registry, comparing PCS to non-PCS groups (19). They found that a prehospital chlorhexidine gluconate (CHG) scrub appears to increase the risk of SSO in patients undergoing ventral hernia repair, suggesting that this is not a desirable measure. After multivariate logistic regression modeling, the preoperative chlorhexidine scrub group had a higher incidence of SSOs (odds ratio [OR] = 1.34; 95% CI 1.11 to 1.61) and SSIs (OR = 1.46; 95% CI 1.03 to 2.07). After propensity score modeling, the increased risk of SSO and SSI persisted (SSO: OR = 1.39; 95% CI 1.15 to 1.70; SSI: OR = 1.45; 95% CI 1.011 to 2.072, respectively). In addition to not being as successful as previously thought, the authors addressed the concern that suboptimal prehospital CHG administration -due to iatrogenic disruption of the existing skin microbiome- may even contribute to bacterial resistance to CHG, or to a possible linkage with antibiotic resistance.

### Surgical Caps

Surgical caps have previously been suggested to influence the incidence of postoperative wound morbidity. Haskins et al. in that matter, compared 6210 cases from 68 surgeons wearing different styles of surgical caps (disposable bouffants, disposable skull caps, cloth skull caps) and found no association between any type of surgical hat worn and the incidence of postoperative wound events (20). A total of 251 (4.0%) patients experienced a postoperative SSI, 743 (12.0%) patients experienced a postoperative SSO, and 361 (5.8%) patients experienced a postoperative SSOPI. This absence of detectable relation with SSO seems to be true for any combination of compared hat types (caps vs bouffant, cloth vs disposable) as well as for ear exposure.

### No-Touch Technique for Mesh Placement

Regarding mesh related morbidity, mesh handling might be of importance. Schneeberger et al. performed a demographic study in 88 patients undergoing ventral hernia repair using a “no-touch” technique during synthetic mesh placement (21). The authors retrospectively reviewed a prospectively maintained database of patients undergoing abdominal wall reconstruction with synthetic mesh from 2013 to 2018 by a single surgeon with a minimum 1-year follow-up. Before placement, the surgical dissection area was copiously irrigated with a triple-antibiotic solution. The “no-touch” technique focused on not removing the mesh from its packaging until immediately before use, to ensure minimal environmental exposure. No contact was made with any instruments, table or sterile drapes. It was dipped in both an antibiotic and a povidone-iodine solution. After placement, the incision was again rinsed with the antibiotic solution. Postoperative complications were observed in 15.9% of patients, of which 6/14 patients (42.9%) were readmitted to the hospital for management. Three of the readmitted patients (3.4%) required reoperations related to abdominal infection and required removal of the synthetic mesh.

This study claimed promising results, with SSI, SSO and recurrence rates below those reported in comparable studies on similar patient cohorts and the authors suggested this no-touch technique for mesh placement might be beneficial in minimizing surgical site occurrences after ventral hernia repair.

### Antibiotic Soak of Mesh Graft

Closely related to the previous “no-touch” technique including the mesh being “dipped in both an antibiotic and a povidone-iodine solution”, Yabanoglu et al. investigated the effect of soaking the synthetic mesh in a vancomycin solution on the rate of mesh infection, compared to a control group exposed to a saline solution soaked mesh (22).The incidence of seroma formation was significantly higher in the group receiving an antibiotic-soaked mesh (3.8% vs. 26.9% respectively, *p* = 0.041). Yet, the overall complication rates were not significantly different between groups. No significant difference was found between the groups in terms of mesh infection.

### Use of Drains

Although traditionally widely used in open ventral hernia repair to prevent seroma and hematoma formation by facilitating fluid drainage, drain placement remains a controversial subject. Several studies in the past have indicated that drains not only fail to prevent seroma formation, but may even contribute to the development of wound infection after ventral hernia repair (37–39).

The effect of drain use on SSI/SSO after ventral hernia repair was investigated in five studies since then, of which four full text versions could be found. They reassessed the relationship between drain usage and postoperative SSI/SSO, as well as important questions regarding the ideal timing of drain removal and the place of concomitant use of prophylactic antibiotics.

Krpata et al. retrospectively reviewed patients after an open ventral hernia repair from the Americas Hernia Society Quality Collaborative (AHSQC) (26). Four hundred eighty-one patients were operated on with drains and 100 without the use of drains. After matching, 300 patients were compared, 200 with drain placement and 100 without. It should be noted that patients with subcutaneous drains were excluded from this study to avoid confounding. In contrast with previous research, the authors found that retromuscular drains were less likely to develop a noninfectious SSO (OR, 0.33) and drain placement was not associated with SSI (OR, 1.30) or SSOPI (OR, 0.94). They concluded that surgical drains do not increase the risk of surgical infectious complications, and may even be protective against some SSOs, such as seroma formation.

Considering timing of drain removal, Plymale et al. retrospectively reviewed a cohort of complex ventral hernia repair cases to determine the incidence of postoperative wound complications and their association to the timing of drain removal (24). All 64 patients included in this analysis were performed by one surgeon and were limited to “clean” wounds (Class 1 CDC classification). Cases were divided into four groups based on duration prior to removal of all drains: ≤7 days (*n* = 18), 8 to 14 days (*n* = 16), 15 to 28 days (*n* = 18), or ≥29 days (*n* = 12). Drains were removed according to predefined standard criteria based on the amount of output (<40 ml/24 h for two consecutive days). No significant relationship was found between incidence of seroma/hematoma and total duration of drainage. However, wound complications were found to increase linearly with time with 10 occasions of SSO (29%) in the patient group that had drains removed in the first two postoperative weeks compared to 17 SSOs (57%) for patients that had drains *in situ* beyond this period (*p* = 0.038).

To compare drains versus progressive tension sutures regarding the incidence of seroma and surgical wound infection following incisional hernia repair, Westphalen et al. conducted a RCT in 42 patients (23). In the drainage group a 4.8 mm diameter continuous closed-suction tubular drain was placed between the aponeurosis and the subcutaneous tissue caudally to the incision. The subcutaneous tissue approximation was performed with separate absorbable sutures. Drains were not used in the second group. Instead, separate absorbable 2–0 polyglactin 910 sutures were placed from the subcutaneous mesh to the aponeurosis every 2 cm by means of the progressive tension suture technique (quilting sutures). Using the quilting suture technique, the frequency of seroma formation did not significantly differ, while the SSI rate was high (21% overall, 4/21 in the drainage group vs. 5/21 in the tension suture group respectively). The authors concluded that drains do not increase the risk of surgical infectious complications as compared to quilting sutures.

Wong et al. investigated the effect of extended postoperative antibiotic prophylaxis with closed-suction surgical drain placement in ventral hernia repair on the incidence of SSI (25). They retrospectively reviewed 234 patients from a single institution who underwent incisional ventral hernia repair. Their results suggested that extended postoperative antibiotics significantly reduced postoperative SSI incidence (OR, 0.31; *p* < 0.01). For hernia grades 2 and above (according to the Ventral Hernia Working Group's hernia grading scale), these findings were confirmed even after stratifying for hernia grading scale as a confounder (OR 0.25, 0.30 and 0.13 for grades 2–3–-4, respectively). This tendency for higher grade hernia patients to benefit more from prolonged prophylactic antibiotics is explained by the authors as a logical result considering that this population is more at risk for SSI development. The authors concluded that their results support the use of extended antibiotic prophylaxis after ventral hernia repair with closed suction drains and encourage implementation in all complex hernia cases (grade 2 and above).

### Prophylactic Negative Pressure Wound Therapy (pNPWT)

The concept of negative pressure wound therapy (NPWT), being a sealed foam dressing through which suction is applied *via* tubing to draw exudate and liquid material from a wound, has been applied in the treatment of difficult wounds that are not suitable for primary closure. More recently its use on closed, primary incisions has been proposed in the prevention of surgical site occurrences. The WHO Global Guidelines described a significant benefit observed in reducing general postoperative infection rates with the use of pNPWT (15). Eight papers were identified in this review that exclusively addressed prophylactic incisional (closed-wound) negative pressure wound therapy after ventral hernia repair. One had to be discarded because a full text was not found (27–33). All studies seem consistent in reporting lower rates of SSI and SSO when using pNPWT (Table 3).

Soares et al. assessed the impact of pNPWT on the outcome of open ventral hernia repair in 199 patients and showed a reduction from 32% to 9% for SSI and from 42% to 17% for SSO in favor of pNPWT (29). However, as a consequence of the pNPWT, hospital stay increased, with 84% of the patients having a length of stay of more than 7 days versus only 39% in the control group (*p* < 0.001). De Vries et al. specifically evaluated SSI and SSO in 66 high risk patients after major and complex ventral hernia repair (30). pNPWT was associated with a significant decrease in postoperative wound infection rate (24 versus 51%; *p* = 0.029). Moreover, SSOPI occurred less frequently in the pNPWT group (*p* < 0.001). Most recently Bueno-Lledo and colleagues showed a reduction of SSOs from 29.8% to 16.6% (*p* = 0.042) comparing 146 patients after ventral hernia repair (33). This was mainly based on a lower incidence of SSI of 0% in the pNPWT group vs. 8% in the control group (*p* = 0.002). They did not see any difference in the rates of seroma, hematoma or wound dehiscence.

### Electric Cauterization of Hernia Sac

This is of less practical use in open ventral hernia repair, unless the hernia sac is used for anterior closure in a bridged repair, but in laparoscopic repair the presence of the hernia sac may be a reason for increased seroma formation. A retrospective propensity score matched analysis by Prassas et al. compared the incidence of seroma formation after laparoscopic IPOM between propensity matched groups of patients with (*n* = 20) and without (*n* = 20) electric cauterization of the hernia sac (34). This measure assumedly eliminates the dead space after hernia repair, by forming adhesions between mesh and cauterized tissue. Cauterization was performed of both the entire hernia sac as well as a surrounding 1 cm rim of peritoneal surface around the hernia defect. There was no resection of the hernia sac, nor closure of the defect. According to their results, cauterization was significantly associated with a reduced rate of postoperative seroma after ventral and incisional hernia repair (0% vs 5% respectively, *p* < 0.05).

### Quilting Sutures

As mentioned previously, the use of quilting sutures aims at obliterating dead space after hernia repair by application of multiple interrupted sutures between the subcutaneous tissue on one side and the underlying sheath and fixed mesh on the other side. Two studies reported on this subject, only one for which a full text was available. A RCT by Alhussini et al. compared seroma formation among 370 patients subjected to ventral hernia repair with (*n* = 190) and without (*n* = 180) absorbable quilting sutures (35). The authors found a significantly smaller amount of output of the drains throughout the hospital stay (every day of the first five postoperative days as well as the total amount of the output before drain removal) in favor of the quilting group (*p* < 0.001). Drains were removed earlier in the quilting group. The incidence of clinically detected seroma in this group was less compared to the control group at all follow-up checkpoints, but was only statistically significant at one week (11.7%) vs. 5 (2.6%); *p* < 0.001).

### Fibrin Sealant (FS)

Azoury et al. compared the incidence of seroma formation and other SSOs in patients undergoing abdominal wall hernia repair, with (*n* = 127) and without (*n* = 123) FS application (36). They aimed to evaluate whether the droplet application of the FS over the entire fascial interface would aid in eliminating post-operative dead space and hence the opportunity for seroma formation. The two cohorts were studied during consecutive time periods. The authors found no advantage for the use of FS in seroma reduction following ventral hernia repair. Moreover, there was a trend towards an increased incidence of seroma in the FS group (FS 11%, no FS 4.9%, *p* = 0.07). An increase for all SSOs was also observed, albeit not statistically significant (FS 18.1%, no FS 13%, *p* = 0.27).

## Discussion

In the current review, a limited amount of evidence was found regarding intra-operative measures to reduce postoperative wound morbidity after ventral hernia repair. Only few randomized controlled trials were found and most studies were retrospective. Although the literature on this topic was sparse, some recent publications indicate increasing interest in this area. In combination with modification of preoperative well-known risk factors as diabetes regulation, smoking cessation and weight loss, a meticulous surgical technique and strategy, general intra-operative tools will help us to improve patient outcomes. Cox and colleagues recently reported increased costs associated with preventable comorbidities in patients undergoing ventral hernia repair, and the AWR Europe collaborative published a consensus statement on perioperative optimization in complex abdominal wall reconstruction (5, 40). SSOs and SSIs are potentially preventable complications that have a substantial impact on the patient and on the cost to the healthcare system. This systematic review including all available literature after publication of the WHO guidelines in 2016 (41) summarizes available evidence on intra-operative measures surgeons could use to optimize outcomes.

According to the WHO recommendation, good clinical practice (GCP) requires that patients bath or shower before surgery (either plain or antimicrobial soap), to ensure that the skin is as clean as possible before surgery and reduce the bacterial load, particularly at the site of incision. Specifically concerning abdominal wall surgery, the results of Prabhu et al. (19) are in contrast with earlier findings, describing either improvement or – such as in a 2015 Cochrane review – no distinguishable advantage with the routine use of preoperative CHG (42–44). A possible explanation suggested by the authors is that these results have captured a more “real world” approximation of how preoperative CHG is actually used – as opposed to a carefully controlled randomized controlled trial, where the results might only be applicable to a narrow and specific population of patients. Lack of standardization of the administration technique may result in a failure of successful skin decolonization. Based on earlier positive results regarding CHG use outside ventral hernia repair surgery, the authors conclude that there might be a subgroup in this specific hernia patient population that can benefit from a standardized use of preoperative CHG. Additional investigation with more directed use of CHG in ventral hernia repair to determine its ultimate effects on wound events is indicated, rather than simply assuming that there is a benefit to using this intervention widely.

Very limited evidence is available regarding the surgical caps or hats used in the operating theaters. Already several decades ago hair was considered a potential reservoir of bacterial commensals that may act as a potential contaminator for surgical sites, leading to increased infections (45). Later on Mase et al. showed firm adherence of Staphylococcus aureus and Staphylococcus epidermidis to human hair, hypothetically leading to SSIs (46). The study by Haskins et al., however, for the first time directly compared the association of surgical hat type with postoperative wound events (20). There is no association between the type of surgical hat worn and the incidence of postoperative wound events following ventral hernia repair. Therefore, without any other evidence available, their findings suggest that surgical hat type may be chosen at the discretion of operating room personnel without fear of detriment to their patients. This is an interesting perspective, countering the predating official recommendations by the Association of periOperative Registered Nurses (AORN) in 2012. The evidence on which these guidelines were based is dated (pre 1980's) and weak at best. Since the current evidence does not demonstrate any correlation between the type of surgical hat and the outcome of SSI rates, the AORN updated their recommendations through the 2020 Guideline Revisions to suggest that an interdisciplinary team at facility level (such as members of the surgical team and infection preventionists) determine the type of head covers that will be worn (47).

When considering the intra-operative setting very little evidence could be identified regarding SSI prevention. An intraoperative no touch technique was proposed by Schneeberger et al., but there were some clear concerns regarding this study (21). Firstly, it is a non-comparative study, designed as a pilot to evaluate the no-touch technique as a benchmark for future prospective studies. More importantly, the authors attribute their results to the no-touch mesh placement technique, while there are many other factors at play. While meticulous mesh handling hygiene may very well be considered GCP, it remains unclear whether this factor on itself will help to reduce the incidence of SSIs.

In strong relation to the no-touch concept, Yabanoglu and colleagues observed the influence of antibiotic (vancomycin) soaking of the mesh before implantation compared to a saline-solution soaked control group (22). This practice was studied before, but mainly in experimental settings (48, 49). Considering that the use of saline wound irrigation is currently under investigation of their anti-seroma properties (50, 51), the potentially positive effects of a saline soak may have had an influence on the findings, providing a possible explanation for the higher incidence of seroma formation in the vancomycin-soaked group. Further prospective comparative studies are needed to confirm the effect of antibiotic soaking, especially considering the different locations of a mesh in ventral hernia repair.

In all areas of abdominal surgery the use of drains is still a matter of debate (52–56). The same is true for subcutaneous and retromuscular drainage after ventral hernia repair. In 2013 a Cochrane review by Gurusamy et al. addressed the scarcity of available evidence on this subject, identifying only one randomized controlled trial (RCT) that evaluated the outcome of drain placement after incisional hernia repair (57). However, this RCT was a comparison of drain types and not a comparison to a control group without drains. Therefore, the Cochrane review concluded there was insufficient evidence for any conclusions to be drawn about the outcome of wound drains after incisional hernia repair.

Krpata et al. showed that retromuscular drainage was negatively associated with noninfectious SSO and not associated with more SSIs (26). They did not only conclude that surgical drains do not increase the risk of surgical infectious complications, but even suggested a protective mechanism against seroma and hematoma formation. These findings are even more striking considering that in the demographic comparison, the patient group receiving drains had greater hernia widths, more complex surgery and longer operative times. Thus, even small simple ventral hernia repairs had higher rates of seroma development than a more complex subgroup of hernia patients that did receive drains. These findings can be explained by fluid accumulation being an important side-effect of surgical tissue dissection. Since fluid might act as an ideal soil for bacterial growth, drainage seems advisable. The duration of drainage has however not been elucidated yet.

Plymale et al. retrospectively showed no significant relationship between timing of last drain removal and the incidence of seroma/hematoma formation (24). However, wound complications were found to increase linearly with time. This may of course not be related to the presence of drains and in contrast, the ongoing fluid drainage might be an indication of other developing wound issues. The 2016 WHO Guidelines advised removing wound drains “when clinically indicated” (15). Based on the available body of evidence at the time, no recommendation around optimal timing of drain removal in the prevention of postoperative SSI can be made.

An additional point of discussion in this debate around the relation between closed-suction drains after VHR and SSI risk, is the precautionary measure of extending postoperative prophylactic antibiotics (pAB). Across all surgical specialties, the 2016 WHO Guidelines recommended against the prolonged use of pAB in the presence of wound drains (15). This recommendation is based on both insufficient evidence to advocate for extended pAB use, as well as the possible harmful effects associated with the practice (such as antibiotic resistance, fungal superinfections and side-effects). Wong et al. suggested, however, that the continued administration of pAB, while drains remain in place, aids in the prevention of surgical-site infections (25). An argument against this practice is the fact that, since evidence of an association between drain placement and SSI development is scant, there is no justification for the use of extended pAB (57). As extended pAB have shown to be protective against SSI in hernia repair studies regardless of the use of closed suction drains (58, 59), the results reported by Wong et al. may be due to the general protective effect of extended pAB for surgical infection without being directly related to the use of drains. In light of the contradictory WHO recommendations, it is clear that higher quality studies specifically targeting the ventral hernia population are necessary to assess efficacy and safety of prolonged pAB before the implementation of this practice should be considered.

For pNPWT the evidence seems stronger regarding the prevention of SSI than for SSOs. In line with the WHO findings, the new evidence confirms that pNPWT appears to be advantageous in ventral hernia repair specifically, as a promising solution to reduce the incidence of SSI. Two separate meta-analyses confirm these findings: Tran et al. found the risk of both SSI and wound dehiscence to decrease by 51% (RR: 0.51) with pNPWT use in abdominal wall reconstruction in high-risk patients. They did not observe a risk decrease for other SSO outcomes (such as seroma, hematoma and re-intervention) (60). Berner-Hansen et al. demonstrated that pNPWT was associated with a decreased risk of both SSO (OR 0.27 [0.19, 0.38]; *p* < 0.001) and SSI (OR 0.32 [0.17, 0.55]; *p* < 0.001). They did not find a statistically significant association with the risk of hernia recurrence (61). While there are many hypotheses as to why pNPWT might have a positive effect on wound healing and SSI/SSO prevention, the precise mechanism remains unknown. Most likely, it is a combination of effective exudate drainage from the wound, which reduces tissue edema without losing an optimal moist healing environment together with mechanical contraction of wound edges and stimulation of blood perfusion in the wound bed, which may contribute to the formation of granulation tissue. A final factor might be protection against micro-organisms from the outside due to the sealed nature of the pNPWT system.

Electric cautery of the hernia sac may have a preventive role against seroma formation following minimally invasive hernia repair. Although the use of medical talc in the subcutaneous space was proposed earlier as an efficient preventive measure (62, 63), later studies regarding onlay mesh repair contradicted those results and no further studies have been reported on this adjunct until now (64). The use of quilt sutures have been reported mainly by plastic surgeons, and is sugested to have a beneficial effect on seroma formation as shown in the RCT by Alhussini et al. (35). They not only found a significantly smaller amount of output of the drains throughout the hospital stay in favor of the quilting group, but drains were removed earlier too. Compared to surgical drains to prevent seroma formation, quilting sutures might have the advantage to provide longer “dead space” elimination than drains, as the peak incidence of seroma formation occurs approximately two weeks after surgery, when prophylactic drains would be useless. While no recommendation around the use of quilting sutures in seroma prevention was made in the 2016 WHO Guidelines (15), the findings of Alhussini et al. seem to be backed up in the literature by other studies (predominantly in populations undergoing abdominoplasty procedures). While evidence is too scant and of insufficient quality to function as the base for any sort of formal recommendation about the use of quilting sutures, these results suggest that they may be an interesting alternative or addition in seroma prevention.

In contrast, the findings for FS in limiting the dead space and seroma formation remain contradictory. The recent report by Azoury and colleagues did not show any effect of FS droplets (36), which contradicts the findings of the systematic review by Morales-Conde et al in 2011 (65). A possible explanation for this result might the difference in amount of sealant as well as different levels of thrombin concentration. Also, the anti-adhesive properties of FS might be less effective when tissue contact is not adequately maintained. In this scenario, the sealant might even function as an anti-adhesive agent and may therefore facilitate fluid accumulation. Noting that the use of a FS is associated with significant costs, additional prospective randomized studies are needed to determine the optimal technique and dosage of FS in ventral hernia repair.

Overall, the current review identified a limited amount of literature reported after the WHO consensus statement in 2016, describing the preventive measures and techniques during abdominal wall reconstruction. This systematic review shows an advantage of closed incision NPWT in prevention of SSI after ventral hernia repair. Despite controversy around the usage of drains, no hard evidence was available to show a causal relationship with SSI/SSO development, provided that drains are not left in place for an excessive amount of time (<2 weeks). It is furthermore suggested that quilting sutures or (in laparoscopic repair) cauterization of the hernia sac may be considered as suitable alternatives or adjuncts to drains in decreasing the incidence of seroma formation. The findings of this review also clearly indicate that tools to decrease SSIs and SSOs after abdominal wall reconstruction continuously need our full attention to improve our patient outcomes and to lower overall costs.

## Data Availability

The raw data supporting the conclusions of this article will be made available by the authors, without undue reservation.
